# Differential evolution algorithm for performance optimization of the micro plasma actuator as a microelectromechanical system

**DOI:** 10.1038/s41598-020-75419-5

**Published:** 2020-11-02

**Authors:** Javad Omidi, Karim Mazaheri

**Affiliations:** Aerospace Engineering Department, Sharif University of Technology, Tehran, Iran

**Keywords:** Renewable energy, Fluid dynamics, Mechanical engineering

## Abstract

Dielectric Discharge Barrier (DBD) plasma actuators are considered as one of the best active electro-hydrodynamic control devices, and are considered by many contemporary researchers. Here a simple electrostatic model, which is improved by authors, and uses the Maxwell’s and the Navier–Stokes equations, is proposed for massive optimization computations. This model is used to find the optimum solution for application of a dielectric discharge barrier on a curved surface of a DU25 wind turbine blade airfoil, in a range of 5–18 kV applied voltages, and 0.5 to 13 kHz frequency range. Design variables are selected as the dielectric thickness and material, and thickness and length of the electrodes, and the applied voltage and frequency. The aerodynamic performance, i.e. the lift to drag ratio of the wind turbine blade section is considered as the cost function. A differential evolution optimization algorithm is applied and we have simultaneously found the optimized value of both geometrical and operational parameters. Finally the optimized value at each voltage and frequency are sought, and the optimum aerodynamic performance is derived. The physical effect of each design variable on the aerodynamic performance is discussed. A design relation is proposed to recommend an optimum design for wind turbine applications.

## Introduction

Dielectric-Barrier-Discharge (DBD) plasma actuators are the least expensive plasma generator in atmospheric conditions. Recently they have been widely investigated to be used in different applications^1–6^, which is of great interest to the industry for many industrial applications, including wind turbine technology. The main advantages of the DBD actuators include their low size, weight, power usage, and fabrication cost. Moreover, since they have no moving part, their maintenance is easy. Physically, these actuators ionize fluid particles, and accelerate them in an electrical field in the fluid flow boundary layer, and energize the whole boundary layer to delay its separation from the boundary surface. To understand, analyze, design and optimize this system, one needs to analyze the interaction between the electrical and hydrodynamic field. Potential of its usage in the wind turbine application for a better efficiency in energy harvesting is very high and is promoted in this article. Due to their extensive usage in different applications, especially new applications^1–9^, we need a better understanding of the effect of different geometrical and operational parameters (e.g. the applied voltage, frequency, and the waveform).

Some of the works are experimentally parametric study, in which the geometrical, material, and also operational parameters were investigated on a flat plate^10–25^. Among these works, Forte et al.^10^ performed an experimental parametric study in order to increase the velocity of the ionic wind induced by such actuators and Thomas et al.^13^ performed an experimental investigation to optimize the body force produced by a single plasma actuator for aerodynamic flow control. A primary goal of this study was to improve the actuator authority for flow control applications at higher Reynolds number than previously possible. Operational parameters in burst mode were investigated for vortex generating by plasma actuator by Xue et al.^23^. Also, the effect of the waveform was considered by Nakano and Nishida^24^. In another work, Grosse and Angland^25^ determined the thrust generation and power consumption of the plasma actuator by an experimental design approach for parametric study.

Also, the effect of the plasma actuator is considered by some other experimental works in combination with optimization algorithms on a curved surface^26–36^. Using an optimization algorithm with multiple input parameters, Matsuno et al.^31^ carried out the optimization of the driving conditions of the plasma actuators by a robust design method for wake control at high dynamic pressure conditions. In another work, Matsuno et al.^32^ applied a Kriging–based genetic algorithm called efficient global optimization. Sulaiman et al.^33^ experimentally used a multi-objective design optimization method to optimize a plasma actuator over an airfoil. A parametric study for flow control application in higher wind speed is done by Hu et al^35^ and waveform applied to the plasma actuator, mounted on a curved surface, was investigated and parametrically studied by Pescini et al.^36^.

Most of the previous studies are experimental, and this usually requires extensive time and resources. On the other hand, new achievements in numerical analysis and exponential increase in the computational power, has made it feasible to have accurate enough simulations of the flow field. The third line of the research in this field is numerical simulation. Some limited works were conducted to study the plasma actuator parameters’ impact on the induced wall jet in a flat plate^37–39^ and its effect on aerodynamic control of the fluid flow in curved surfaces^40–42^.

Utilizing the electrostatic model some of the geometrical and operational parameters of the plasma actuator is analyzed by Seth et al.^38^. Regis de Quadros et al.^39^ investigated the possibility of the plasma actuator effect on wave control in a flat surface boundary layer and optimized this effect. For reducing the power consumption, Sato et al.^40^ conducted a complete parametric study with several large eddy simulations of the airfoil separated flow control by DBD plasma actuation. Also, to introduce a guideline for the plasma actuator design in fluid flow control, a many-objective optimization on four plasma actuator parameters is studied by Watanabe et al.^41^. In another numerical work, Williams et al.^42^ presented a quantitative design optimization approach for maximizing the fluid flow control authority.

Computational analysis of the real physics of plasma actuators is very complicated, involving interactions of ionization, fluid flow and the electrical field. Accurate solution requires simultaneous solution of the Maxwell and Navier–Stokes equations. There are few attempts to solve this complicated non-linear combination with some assumptions^43–52^. One of these attempts was presented by Suzen and Huang^47^. Basically, they introduced an electrostatic model (S–H model), based on imperial formulation and relations^51,52^ to simulate the plasma actuator effect on fluid flow. Based on experimental results, they assumed a one dimensional Gaussian distribution for the charge distribution over the dielectric surface^52^. The main feature of this model is its simplicity for modeling the plasma actuation, however this model has some drawbacks which the most important one was dependency of this model to the experimental results for calibration. Many improvements to the S–H model were presented, including ones proposed by Bouchmal^48^, Skote et al.^49^, Abdollahzadeh et al.^50^, and Omidi and Mazaheri^3,44,45^. Also, in recent works, Amanifard et al.^53^ used an explicit model to prescribe the wall jet and add it as a boundary condition to the hydrodynamic simulation for fluid flow over a flat plate. Lilley et al.^54^ presented a model to find the forcing function induced by a channel plasma actuator, which may be used in a 3D hydrodynamic simulation code, and they experimentally validated their model.

Authors have used a combination of different numerical models to propose a new model based on Suzen and Huang^46,47^. This proposed model allows the S–H model to consider the effect of voltage and frequency on actuator performance^44,45^. This is applied to a wind turbine airfoil and the effect of voltage, frequency and position of actuators on its performance is investigated. Authors have also proposed an improved phenomenological electrostatic model which is independent of the experimental data^45^. Also, in another work, the authors provide a tool for generating more power by a 6 MW wind turbine full-scale blade^3^.

The lack of an effective numerical model in this field is evident. To address the lack of this comprehensive study, here we use the improved electrostatic model^44,45^, which is validated and tested on real wind turbine applications^3^. We use the differential evolution optimization algorithm and define an optimization problem to optimize the aerodynamic performance. This is used to find the design variables for the optimum performance of the DBD plasma actuators for a wide range of frequencies and voltages. Also, an engineering formula is proposed to be used in design of optimum wind turbine blades.

Our main contributions in this paper include:

- Usage of a phenomenological model of a plasma actuator for optimization applications.
- Definition of an optimization problem for application of plasma actuators on wind turbine blades.
- Usage of differential evolution algorithm to find the optimum solution for plasma actuators.
- Proposing a new engineering formula for design of plasma actuators for wind turbine blades.

## Governing equations

### Electrostatic model

The electrostatic model proposed by Suzen and Huang^46,47^ computes a Lorentz body force by solving two elliptic equations. The components of this body force are added to the momentum equations as a source terms to incorporate the effect of the plasma actuator. The most important feature of this model is its simplicity to model the interaction of the plasma actuator with the fluid flow. This feature makes it possible to have a model suitable for engineering analysis with sufficient accuracy and with a very low computational cost. In fact, users of this model do not seek the details of the generated plasma and its spatial and temporal structures in the alternating voltage cycles, but to study the overall effect of a plasma actuator on fluid flow control.

This model is one of the most physical models for applying the effect of AC plasma actuator on fluid flow^55^. However, there are two main drawbacks to this model. The first is that the charge density of the generated plasma is independent of the applied voltage. This problem has been solved by an improved model developed by authors^45^ using a boundary condition between the density charge and the electric field. The second drawback is that most parameters involved in the model need to be calibrated with experimental data for each new geometry and operational condition, e.g. different applied voltages. Many people have tried to remove this deficiency^44,45,48–50,53^. To solve this problem, authors proposed some analytical and semi-empirical relationships for the parameters which do not need for further calibration for different geometries, or applied voltages and frequencies. In this section, the basic equations of the electrostatic model and the improvements made to the model by the authors which are used in this work are presented. More details are given in^45^.

By imposing a high AC voltage on an arrangement of two electrodes separated by a dielectric layer, the adjacent air starts to ionize unsteadily and a small plasma region is formed. This continuous plasma formation process happens in a time scale of 10^–9^–10^–8^ s^48^, while the response time of the neutral flow affected by the plasma induced wall jet in most real applications is in the order of 10^–2^ s. Presence of ionized particles in the electrical field results in a body force that acts on the flow in a quasi-steady manner. The Electro-Hydro-Dynamic (EHD) body force is due to the momentum transfer from the charged particles to the neutral ones through collisions. As described in details in^56,57^, the true EHD body force is comprised of linear and nonlinear terms. The linear terms include the conduction terms of the current densities, the diffusion, and the charged particle generation^56,58^. The non-linear term which is recently discussed by Mahdavi and Sohbatzadeh^57^, depends on the gradient of the electric field intensity and the variation of the plasma dielectric permittivity. Here, by neglecting the magnetic forces and based on other assumptions that are used in this model^45^, the true term of the body force is simplified to the well-known EHD body force:

1 $$\overrightarrow{{f}_{b}}={\rho }_{c}\overrightarrow{E}$$

where, $\overrightarrow{{f}_{b}}$ is the body force in $N/{m}^{3}$, ${q}_{c}$ is the net charge density in $\mathrm{C}/{\mathrm{m}}^{3}$, and $\overrightarrow{E}$ is the electric field vector. Suzen et al.^46,47^ assumed that the extension of the charged area normal to the surface, i.e. the Debye length, is small and one may presume that the electrical interaction of the charged particles is limited to the electric charge on the wall. Although the external electrical field is varying, the potential made by the electric charge on the wall may be assumed unchanged. To estimate the value of the body force, they assumed that the gas particles are weakly ionized and there is enough time for their redistribution to consider the process to be quasi-steady, and the interactions are weak enough to use the superposition of the induced potential of the external electrical field and the net charge density. This directed them to simplify the Maxwell equations and use two independent equations for distributions of the potential electric field and the charge density as:

2 $$\nabla .\left({\varepsilon }_{r}\nabla \phi \right)=0$$

3 $$\nabla .({\varepsilon }_{r}\nabla {\rho }_{c})=\frac{{\rho }_{c}}{{\lambda }_{d}^{2}}$$

where $\phi$ is the electric potential, ${\lambda }_{d}$ is the Debye length, and ${\varepsilon }_{r}$ is the relative permittivity.

#### Modified boundary conditions

**Figure 1:**
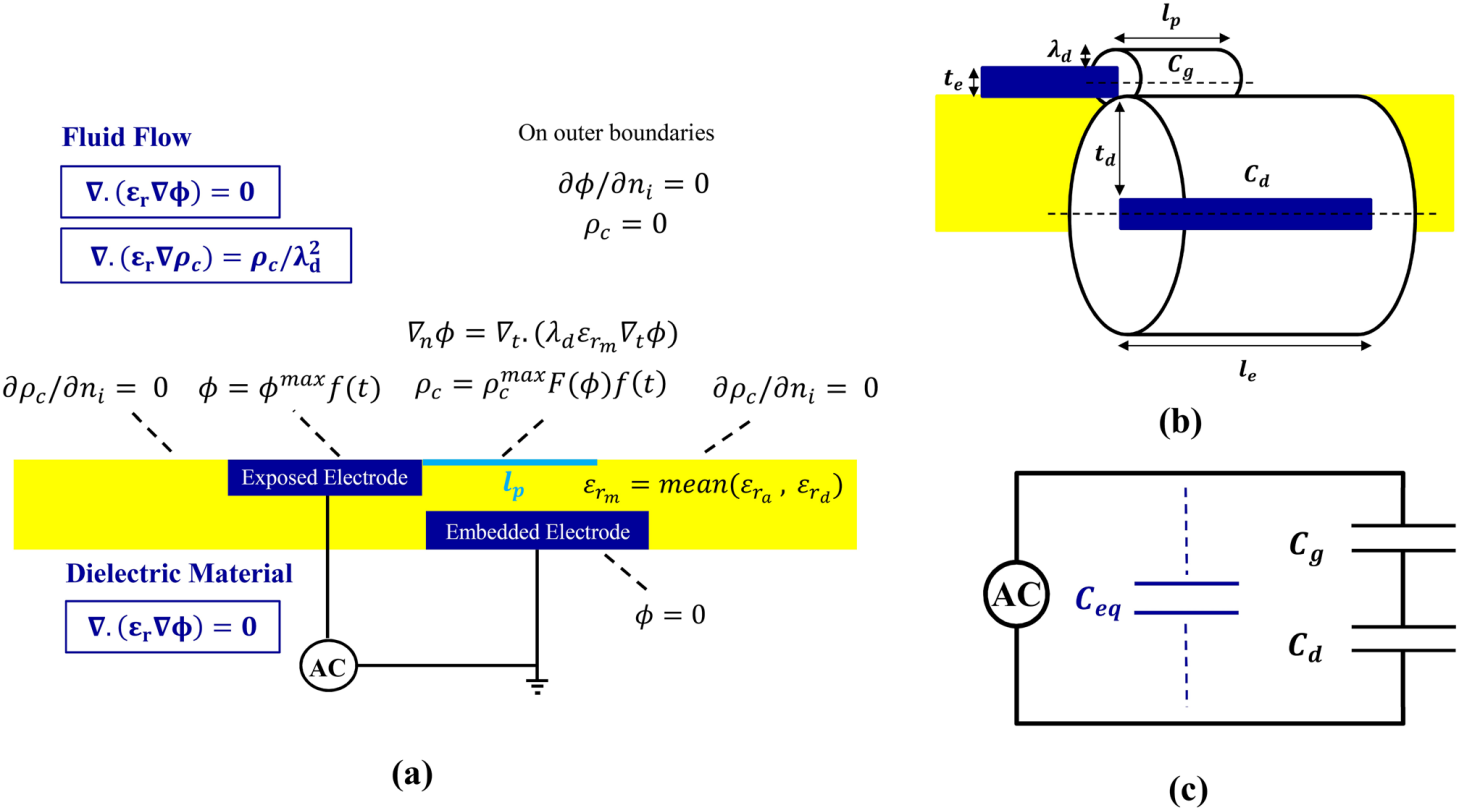
The schematic of a DBD plasma actuator (**a**) boundary conditions for the electrical distribution and the density charge, (**b**) cylindrical capacitors for the improved model, (**c**) equivalent circuit of capacitors.

These two differential equations need to two different sets of boundary conditions to be solved. The electric potential Eq. (2) is solved in both the dielectric and the fluid domain (Fig. 1a). Boundary conditions are defined as: $\partial \phi /\partial {n}_{i}= 0$ on outer boundaries, $\phi =\phi (t)$ on the exposed electrode, and $\phi =0$ on the embedded electrode. Here ${n}_{i}$ is the unit vector normal to the surface and $\phi \left(t\right)={\phi }^{max}f(t)$ denotes the applied voltage. ${\phi }^{max}$ refers to the amplitude of the applied AC voltage. The wave form function $f(t)$ is a time-dependent function.

The net charge density Eq. (3) is solved only on the fluid flow side of the domain (Fig. 1a). Boundary conditions for solution of this equation are given as: ${\rho }_{c}=0$ on outer boundaries, ${\rho }_{c}={\rho }_{c}^{max}G\left(x\right)f\left(t\right)$ for downstream of the exposed electrode, on the surface of the dielectric above the embedded electrode, and $\partial {\rho }_{c}/\partial {n}_{i}= 0$ on the solid walls except the region of the embedded electrode. ${\rho }_{c}^{max}$ refers to the maximum charge density of the applied AC voltage on the dielectric surface. Based on the experimental results^52^, Suzen and Huang^46,47^ suggested a half Gaussian distribution to simulate the variation of the charge density on the wall over the embedded electrode in the stream-wise direction. ${\lambda }_{d}$ and ${\rho }_{c}^{max}$ remain to be determined later by an empirical or phenomenological model.

Like any other phenomenological model, the S–H model has its shortcomings. The charge density computed by this model is independent of the applied voltage, which is due to the decoupling of the governing equations. Also the simulated induced wall jet is thinner than the real boundary layer^47^. This results in a boundary layer velocity profile very different from experimental data^47,49^. The overall accuracy of the simulated boundary layer predicted by the S–H model is very poor. For more accurate simulation of the electrical potential Ibrahim and Skote^49^ presented a new boundary condition for solving the Eq. (2) at the charge surface:

4 $${\nabla }_{n}\phi ={\nabla }_{t}.({\lambda }_{d}{\varepsilon }_{r}{\nabla }_{t}\phi )$$

where ${\nabla }_{n}$ and ${\nabla }_{t}$ describe the normal and tangential derivatives in respect to the surface.

To resolve this problem, we have introduced a new boundary condition for the charged surface to be used instead of the Gaussian distribution^44,45^. In the new boundary condition the distribution of the charged density on the electrode boundary surface is proportional to the electric potential. The solution procedure follows. First Eq. (2), i.e. the electric potential equation is solved to result in the distribution of the potential electric field on the charged surface. Then, to find the boundary condition for the net charge density Eq. (3) along the plasma extent, this value is used in Eq. (5).

5 $$\begin{aligned} & 0 < x < 17\% \, \rho_{c} \left( x \right) = \rho_{c}^{max} \left( {\frac{{\phi_{max}^{local} - \phi }}{{\phi_{max}^{local} - \phi_{{17_{\% } }} }}} \right)^{2.0} \\ & 17\% < x < 100\% \, \rho_{c} \left( x \right) = \rho_{c}^{max} \left( {\frac{{\phi - \phi_{min}^{local} }}{{\phi_{{17_{\% } }} - \phi_{min}^{local} }}} \right)^{0.6} \\ \end{aligned}$$

#### Semi-empirical parameters involved in the model

We assume that the DBD actuator induces a thrust force proportional to the consumption of the electrical energy^50^. One may use the schematic AC circuit (Fig. 1b,c). to calculate the energy consumption, and therefore to find an accurate estimate of the thrust. This was introduced by Yoon and Han^37^, to model a DBD actuator by an AC circuit with two serially connected capacitors. They used the consumed energy of the capacitors to estimate the value of thrust. ${C}_{d}$ is the capacitance of the combination of the embedded electrode and the dielectric region, while the capacitance of the exposed electrode and the generated plasma over the electrodes is ${C}_{g}$ (Fig. 1b). One may approximate the capacitance of the capacitors based on the geometric and material properties of the actuator components, as was described by Yoon and Han^37^:

6 $${C}_{g}=2\pi {\varepsilon }_{0}\frac{{l}_{p}}{\mathrm{ln}\left(\frac{0.5{t}_{e}+{\lambda }_{d}}{0.5{t}_{e}}\right)}$$

7 $${C}_{d}=2\pi {\varepsilon }_{d}\frac{{l}_{e}}{\mathrm{ln}\left(\frac{0.5{t}_{e}+2{t}_{d}}{0.5{t}_{e}}\right)}$$

Here, ${t}_{e}$ is the thickness of the exposed electrode, ${t}_{d}$ is the thickness of the dielectric barrier, ${l}_{p}$ is the length of the plasma extent on the dielectric surface, ${l}_{e}$ is the length of the embedded electrode, ${\varepsilon }_{0}$ is the permittivity of the free space and ${\varepsilon }_{d}$ is the dielectric permittivity.

To find the Debye length over the dielectric surface, Bouchmal^48^ has solved an inverse problem using the S–H model, and the experimental data of Kotsonis et al.^59^ for body forces to find the charge density distribution over the flow domain. This data is valid for a range of applied voltages with a frequency of 2 kHz. According to Bouchmal^48^ the Debye length is considered as a linear function of the applied voltage:

8 $${\lambda }_{d}\left[m\right]=0.2\left(0.3{\times {10}^{-3}V}_{app}-7.42\times {10}^{-4}\right)$$

where ${V}_{app}$ is the applied voltage in kV. The dependence of the Debye length on frequency is ignored in this equation.

Kotsonis et al.^59^ observed that this length is generally a function of both the applied voltage and frequency, but for high enough frequencies the dependence on frequency is negligible^10^. When we used an inverse analysis to find the charge distribution from body forces, we found that the Debye length is a function of the frequency, as well. As described in^45^, a curve fit to the experimental data^59^ results in Eq. (9), which is used as a correction factor for Eq. (8):

9 $${\alpha }_{{\lambda }_{d}}\left(f\right)=0.5611Arctan\left({-170.3\left(f\right)}^{-5.124}\right)+1.768$$

In this equation frequency is in kHz. One of the most important parameters in the simulation of the plasma actuator performance is the breakdown voltage, at which the first ionization occurs. Here we use the Peek’s experimental law to find this critical voltage. Yoon and Han^37^ modified this equation for plasma actuators by adding four parameters to the equation. These parameters are ${t}_{d}$, ${t}_{e}$, $P$, the air pressure and $T$, the air temperature. The Peek’s law is modified for the range of 3–35 kV voltage and 1–14 kHz frequencies, to result in:

10 $$\begin{aligned} & V_{bd} \left[ {kV} \right] = m_{v} g_{v} \left( {\frac{{t_{e} }}{2}} \right)\ln \left( {\frac{{2t_{d} + 0.5t_{e} }}{{0.5t_{e} }}} \right) \\ & m_{v} = 1\;\;\;\;\left( {\text{smooth surface}} \right) \\ & g_{v} \left[ {kVcm^{ - 1} } \right] = 31\delta \left( {1 + \frac{0.308}{{\sqrt {0.5\delta t_{e} } }}} \right) \\ & \delta = \frac{0.386P}{{273 + T}} \\ \end{aligned}$$

In our previous work^44^ we have derived an algebraic equation to predict the plasma extent:

11 $$\begin{aligned} & a_{1}^{2} l_{p}^{5} + 2a_{1} a_{2} l_{p}^{4} + a_{2}^{2} l_{p}^{3} = a_{3}^{2} \\ & a_{1} = 16000C_{g0} \\ & a_{2} = 16000C_{d0} l_{e} \\ & a_{3} = \sqrt \rho fC_{g0} C_{d0} l_{e} \left( {V_{app} - V_{bd} } \right)^{2} \\ \end{aligned}$$

where ${l}_{p}$ is the plasma extent in m, ${l}_{e}$ is the embedded electrode length in m and, $\rho$ is the density of air in kg/m^3^. We define ${C}_{{g}_{0}}={C}_{g}/{l}_{p}$ and ${C}_{{d}_{0}}={C}_{d}/{l}_{e}$.This equation shall be solved numerically by the classic Newton–Raphson method. To modify this model to be consistent with other model modifications presented, a new corrected value for the plasma extent was introduced by authors in^45^.

Finally, since the maximum charge concentration is a very important parameter in the present model, a modified equation to estimate the maximum charge density in terms of the induced thrust was proposed in^45^. Based on the charge variations along the length of charge development, a correction factor was implemented there:

12 $${\rho }_{c}^{max}={\alpha }_{corr}\frac{Thrust}{{\lambda }_{d}({V}_{app}-{V}_{bd})}$$

where ${V}_{bd}$ is the breakdown voltage, i.e. the least voltage required to start the ionization process^45^.

### The non-dimensionalized form and the numerical simulation process

Non-dimensionalization of the governing equations helps to simplify implementation of the boundary conditions and also to reach a better understanding of the results. Equations (2) and (3) are time independent, but the boundary condition of Eq. (2) for the exposed electrode, and also the boundary condition for Eq. (3) for the charging surface boundary conditions are time dependent. We assume that the time dependence of the boundary conditions are decoupled from hydrodynamic features of this flow field, therefore one may remove the time dependence of the applied voltage by a non-dimensionalization procedure. The resulting non-dimensioanalized or normalized parameters are defined by relations (13). Application of this normalization process to the governing equations of the electrostatic model, results in the non-dimensionalized Eqs. (14) and (15).

13 $$\begin{aligned} & \phi^{*} = \phi /\phi^{max} f\left( t \right) \\ & \rho_{c}^{*} = \rho_{c} /\rho_{c}^{max} f\left( t \right) \\ & \vec{E}^{*} = \frac{{\vec{E}}}{{E_{0} }} = l_{p} \nabla \phi^{*} , \\ & E_{0} = \frac{{V_{app} - V_{bd} }}{{l_{p} }} \\ \end{aligned}$$

14 $$\nabla .\left( {\varepsilon_{r} \nabla \phi^{*} } \right) = 0$$

15 $$\nabla .\left( {\varepsilon_{r} \nabla \rho_{c}^{*} } \right) = \frac{{\rho_{c}^{*} }}{{\lambda_{d}^{2} }}$$

Two-dimensional incompressible Reynolds-Averaged Navier–Stokes (RANS) equations are employed for fluid flow simulation^44^. Since most of the energy given to the plasma actuator is consumed to accelerate the fluid particles and only a negligible part is contributed to the fluid warming^60^, the flow field energy equation is ignored. The fundamental equations of mass and momentum conservation that are used to simulate the fluid flow are as follows:

16 $$\nabla .\overrightarrow{u}=0$$

17 $$\left(\overrightarrow{u}.\nabla \right)\overrightarrow{u}=-\frac{1}{\rho }\nabla P+\upsilon{\nabla }^{2}\overrightarrow{u}+\overrightarrow{{f}_{b}}$$

in which $\overrightarrow{{f}_{b}}$ is the body force per unit volume in $\mathrm{N}/{\mathrm{m}}^{3}$ due to the effect of plasma actuator. $\overrightarrow{u}$, $\rho$, $P$ and $\upsilon$ are respectively, the velocity , the density, the static pressure and the kinematic viscosity. As seen in Eq. (17), the body force generated by the plasma actuator is added to the right hand side of the momentum equation. Solving these equations and simulating of the fluid flow with direct interaction with the electrostatic field is achieved using the finite-element-based computational package COMSOL Multiphysics 5.2. This solver is capable of simultaneous handling of interaction of two different physics (electrostatic and fluid dynamics). Also, the Differential Evolution (DE) algorithm code is developed in C++ language to implement the optimization process linked to the fluid flow solver.

### Geometrical modeling of the plasma actuator

**Figure 2:**
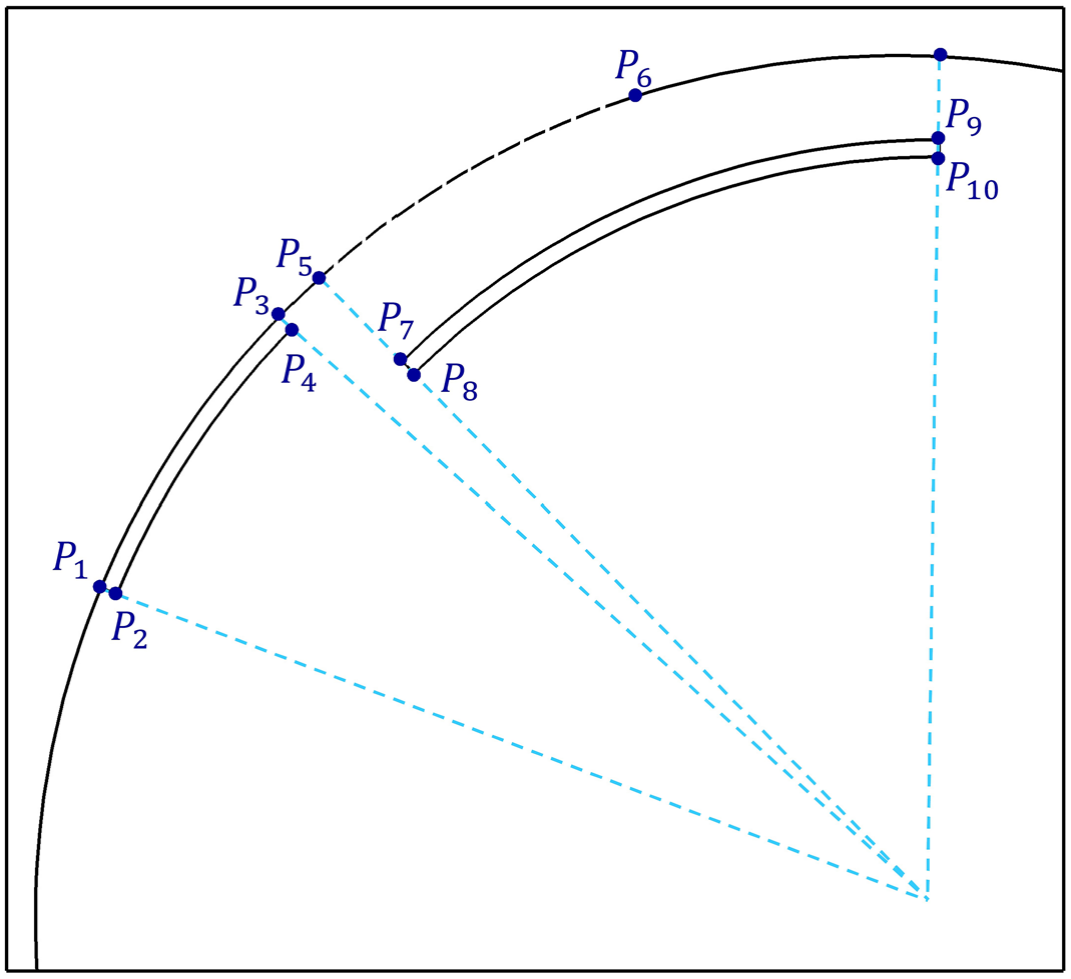
The schematic of the geometric model of the plasma actuator used in the optimization process.

Figure 2 shows our geometrical modeling of the flow domain. Points 1–4 define vertices of the exposed electrode, points 5–6 define the charge surface, and points 7–10 define vertices of the embedded electrode. Other points are also used as minor points, to specify the flow solution domain. To design the geometry of the plasma actuator in each stage of the optimization process, seven values are sufficient to locate all these points. In our optimization procedure, we only use three values, which are described later. The airfoil surface is modeled by an arch. Bezier functions is used to model the surface of the DU25 airfoil^61^. Using differentiation relations, the lines normal to boundaries are found. The electrode surface is parallel to the airfoil surface. Here the actuator location (location of p3) is fixed at 30% of the chord^44^. All dimensions are varied respect to this reference point.

### Differential evolution (DE) optimization

The differential evolution optimization scheme was first introduced by Storn and Price^62^ to fit a polynomial to the Chebychev function. This non-gradient optimization scheme showed very effective to find the extremums in continuous spaces, and therefore has been widely used in scientific and engineering applications^63^. This algorithm is somehow similar to both random search (genetics based) optimization algorithms and gradient based schemes that compute a gradient to find the maximum slope direction in the objective function domain. Instead, to find the extremum, they use subtraction of two vectors that simulates the gradient computation. This model has shown to be capable to find the global extremum with a high speed and accuracy.

The optimization process is made up of four main steps:

- Random generation of the first generation: Each generation has a preselected number of population, and its attributes (gens, design variables) prescribe a vector.
- Mutation step: This process returns the lost gens or undiscovered gens to the generation and prevents convergence to the local extremums.
- Recombination step: The combination of the current population with the mutated population provides a random structural interaction, and provides a possibility of generation of better people from the previous best people.
- Selection step: Comparison of the current population and the recombined people, and using the natural selection principles, the best people remain, and weaker solutions are deleted.

### The optimization problem

To define a new optimization problem, three different components shall be determined: The objective function (to be minimized), the design variables (independent variables of the objective function), and their constraints. Here we select the ratio of drag to lift coefficient of the blade airfoil section as the objective function to be minimized. Design variables include three geometrical values, and one physical property, i.e. the dielectric permittivity of the dielectric material. Table 1 shows the design variables, and their numerical constraints. The length of the exposed electrode is found to be less effective, and therefore is not selected as a design variable. In this study, we have solved the optimization problem for fifty different cases (i.e. 5 different voltages: 7, 9, 11, 13, 15 kV, and 10 different frequencies from 1–10 kHz). Here we have assumed the mutation factor $F=0.5$, and ${C}_{r}=0.8$, ${N}_{p}=5$. If convergence is achieved at generation 50, it requires 250 times solution of the electrostatic and hydrodynamic equations in our grid.

**Table 1:** Optimization constraints of the design variables.

Variables	Minimum (SI)	Maximum (SI)
${l}_{e}^{emb}$	0.001	0.055
${t}_{e}$	0.000005	0.001
${t}_{d}$	0.00005	0.015
${\upvarepsilon }_{\mathrm{rd}}$	0.5	11

### The optimization algorithm

First we define an objective function to be minimized, which is a function of D design variables. The first generation has ${N}_{p}$ population, which is usually 5 to 10 times our design variables D. In an application like ours, in which computation of the objective function is very time consuming, the lower value is selected. Each member $i$ of the generation *G* is presented by a vector ${x}_{i,G}$ in a D-dimensional space [Eq. (18)]. Each entry of this vector is one attribute (gen) of this member, and each design variable is limited to its lower and upper limits, by Eq. (19).

18 $${x}_{i,G}=\left[{x}_{i,G}^{1}, {x}_{i,G}^{2}, \dots , {x}_{i,G}^{D}\right], i=1, 2, \dots , {N}_{p}$$

19 $${x}_{min}^{j}\le {x}_{i, 1}^{j}\le {x}_{max}^{j}, j=1, 2, \dots , D$$

In the mutation step to generate a new member corresponding to *x*_*i,G*_, three different members of the current population are randomly selected, namely, ${x}_{{r}_{1},G}$, ${x}_{{r}_{2},G}$, and ${x}_{{r}_{3},G}$, where $i$, ${r}_{1}$, ${r}_{2}$, ${r}_{3}$ are different numbers. According to Eq. (20), a new vector is made up of these three, i.e.

20 $${\vartheta }_{i, G+1}={x}_{{r}_{1},G}+F\left({x}_{{r}_{2},G}-{x}_{{r}_{3},G}\right)$$

where *F* is called the mutation factor, which determines our step size in the evolution direction $\left({x}_{{r}_{2},G}-{x}_{{r}_{3},G}\right).$ *F* is a number usually selected between 0 and 2. If we have many closely located extremums, a small *F* is more appropriate to find the absolute extremum, but it is too time consuming. For small mutation factors, more dense population is required to make the computation sensible. In aerodynamic design problems, we usually select a mutation factor between 0.4 and 1.

In the next step, i.e. recombination, a test vector ${u}_{i,G+1}$ is generated, whose components are a random selection of components of ${\vartheta }_{i,G+1}$, and ${x}_{i,G}$ as shown in Eq. (21). This random selection is based on a number ${C}_{r}$ , which is between 0 and 1, and determines how much a new member inherit its attributes from its parent ${x}_{i,G}$ or other members of the last generation. Therefore, for each design variable (dimension) $j$, a random number between 0 and 1 is generated, and if this is less than *C*_*r*_, attributes of other members are inherited, instead of its parent. To guarantee that the old member is not reselected, we also generate an integer random number ${I}_{rand}$ between 1 and D, and at least the ${j}^{th}$ attribute is inherited from other members (${u}_{i,G+1}\ne {x}_{i,G}$ and ${\vartheta }_{i,G+1}\ne {u}_{i,G+1}$).

21 $$u_{i, G + 1}^{j} = \left\{ {\begin{array}{*{20}l} {\vartheta_{i,G + 1}^{j} } & {if \; Rand_{j} \left[ {0,1} \right) \le C_{r } \; or \; j = I_{rand} } \\ {x_{i,G + 1}^{j} } & { if \; Rand_{j} \left[ {0,1} \right) > C_{r } \; and \;\;j \ne I_{rand} } \\ \end{array} } \right.$$

The last step is a comparison between the target (parent) and the test vector [Eq. (22)], to select that one which corresponds to the lowest (or highest) objective function.

22 $${x}_{i,G+1}=\left\{\begin{array}{l}{u}_{i,G+1} \quad \;if \;\; f({u}_{i, G+1})\le f\left({x}_{i,G}\right)\\ {x}_{i, G} \quad \quad otherwise\end{array}\right.$$

**Figure 3:**
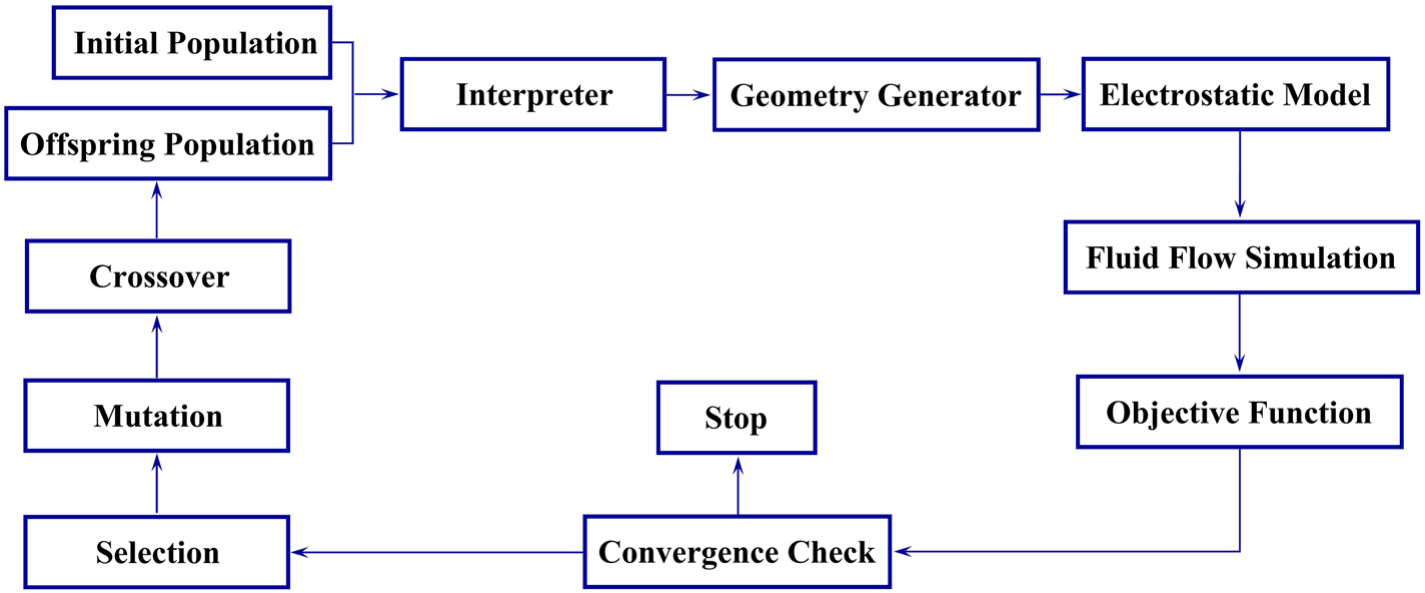
The optimization algorithm flowchart.

The optimization algorithm flowchart is depicted in Fig. 3. It has three main steps. First, ${N}_{P}$ candidates are randomly generated within the constraints of Eq. (19) for the first generation. Each candidate represents a unique configuration of the DBD plasma actuator. Each configuration has unique design variables, and each design variable is selected based on the optimization constraints. At the second step, based on the geometrical model of the actuator and the blade, the grid inside the blade (dielectric material) is regenerated. Then, the charge density and electrical potential are computed using the electrostatic model and by solving the Navier–Stokes equations, the objective functions are computed for each member of the population. At the third step, results are analyzed by the optimization algorithm, and considering the optimization constraints, a new generation of candidates is created. The last two steps are repeated until the solution converges to a final solution.

## Validation of the simulation and optimization

### Fluid flow control over a flat plate

Activation of the plasma actuator induces an electrical field in the nearby fluid, and also the high AC voltage, ionize the adjacent fluid particles, which are accelerated in the electrical field, and their high momentum is transferred to the fluidic region in the boundary layer which results in an induced wall jet. This physics is described in details by authors^44^. Here we investigate the accuracy of the proposed algorithm to simulate the interaction of the DBD induced wall jet with the boundary layer over a flat plate for a stagnant air condition. This has been experimentally investigated by Thomas et al.^13^, using two different DBD configurations of single and tandem actuators. Although a simple model, this is believed to be a good bench mark for validation of numerical schemes, and to evaluate the strength of the simulated induced wall jet, and its interaction with the adjacent fluid. Applied voltage and frequency are respectively 24 kV and 2 kHz. The length of the exposed electrode and embedded electrodes are 10.16 cm and 2.54 cm, respectively. The thickness of the electrodes are 40 μm and the dielectric is made of Teflon with 6.35 mm thickness and relative permittivity of 2.

The numerical domain is a rectangle by 1 m in length and 0.5 m in height. The lower boundary condition is no slip, and the symmetry condition is applied to the upper boundary. The velocity in the left inlet boundary is set to zero. The actuator is located near to the inlet boundary. A stretched structured grid with a high density close to the lower boundary (the actuator location) is used. The height of the smallest grid cells is thirty times smaller than the Debye length, to be able to resolve most of the hydrodynamic details^48^.

**Figure 4:**
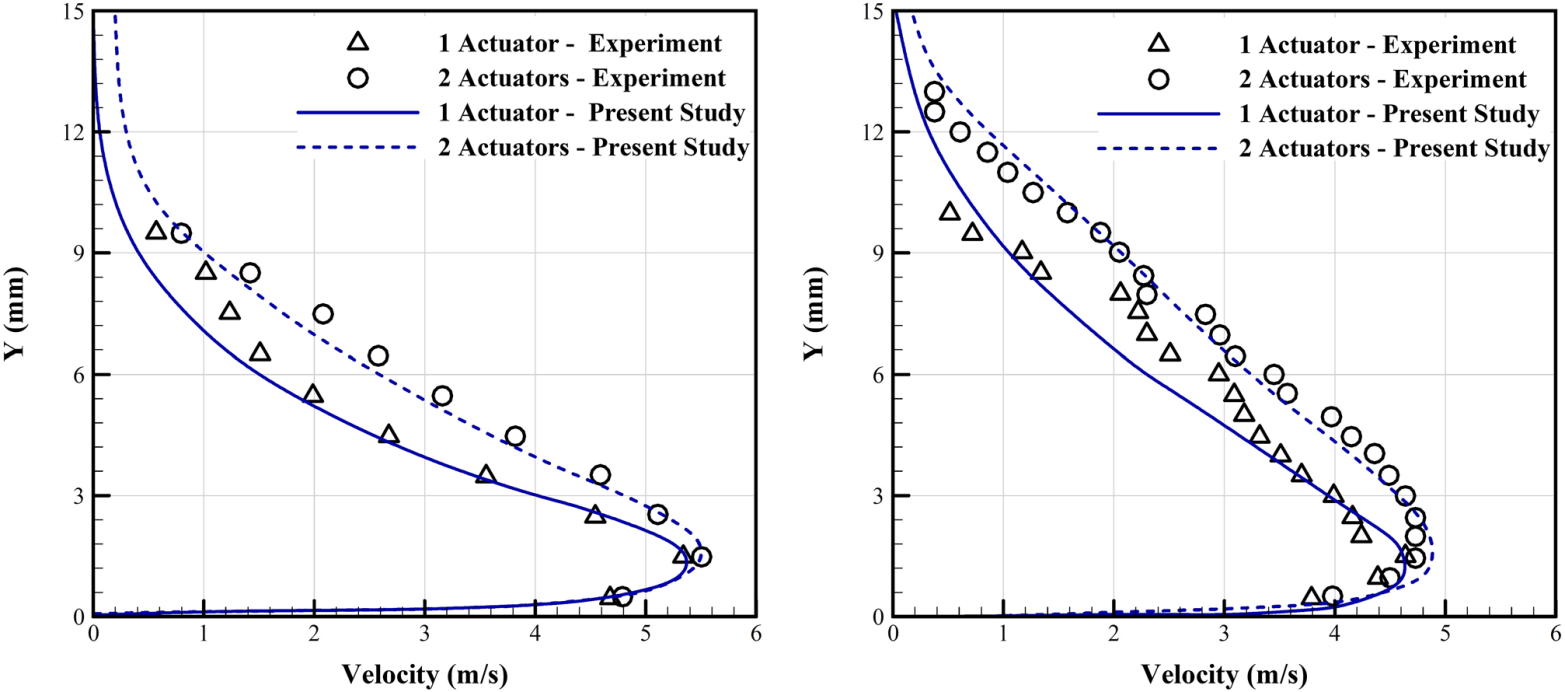
Comparison of the numerical and experimental results^13^ of the induced velocity profiles over a flat plate with stagnant air at two sections, Left: at 3.81 cm, Right: at 5.08 cm downstream of the trailing edge of the exposed electrode. (For the numerical simulation, the material and geometry of the plasma actuator and other aerodynamic conditions are selected similar to the experiment, V = 24 kV, f = 2 kHz).

Figure 4 compares the simulated induced velocity profiles with the experimental results^13^ at two different sections downstream the trailing edge of the exposed electrode. The numerical scheme is well accurate to simulate the jet induced boundary layer thickness, and maximum velocity. The relative error in computed maximum velocity is less than 5% at both sections.

### Fluid flow control over an airfoil

To study validation of the proposed algorithm for simulation of interaction of the plasma actuator with the flow around an airfoil, the experimental results of Wang et al.^64^ are used here. They used a NACA0015 with a chord of 100 cm, an angle of attack of 16°, and a freestream velocity of 20 m/s. The actuator is located at the leading edge, and a harmonic voltage with different frequencies is applied to the exposed electrode. The length of the exposed electrode is 440 mm, the thickness is 0.05 mm, and the dielectric thickness is 0.1 mm, which is made of Kapton. The embedded electrode is ground to zero voltage. The applied voltage is 2.4 kV and the frequency is 3 kHz. SST transition turbulence model is used here to accurately resolve details of the local turbulence flow. The computational grid is a multi-block mesh, details of which are given in reference^45^.

**Figure 5:**
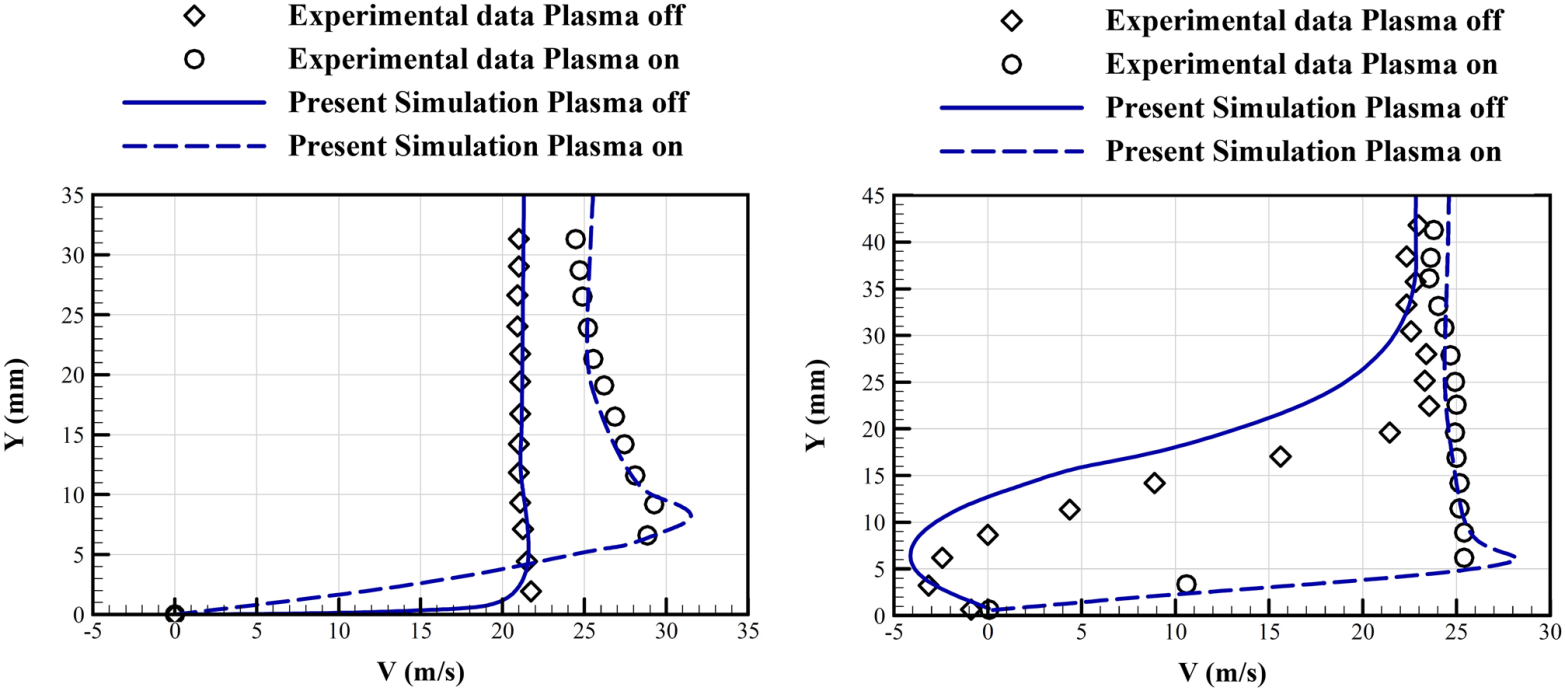
Comparison of the computed boundary layer velocity profiles with the experimental data^64^ for the plasma actuator off and on. Left: at the 10% chord section, Right: at the 40% chord section (from the leading edge). (For the numerical simulation, the material and geometry of the plasma actuator and other aerodynamic conditions are selected similar to the experiment, NACA0015 airfoil, freestream velocity 20 m/s, 16 degree angle of attack, V = 2.4 kV, and f = 3 kHz).

Figure 5 shows the boundary layer velocity profiles at two different sections, at 10% and 40% of chord length, normal to the airfoil surface. Comparisons with the experimental results are performed for two conditions, with the plasma actuators on and off. Again we observe an acceptable validity for the numerical results. In the case with the actuators on, the numerical results predicts a higher velocity, which may be due to our higher resolution in comparison with the experimental facility. The lift coefficients were also predicted with an acceptable accuracy (not reported here).

### Validation of the optimization algorithm

To examine merit of the optimization algorithm, the well-known Schwefel function is used. We use the differential evolution algorithm to find the absolute extremum of the two dimensional version of this function which has many local extremums:

23 $$f\left( x \right) = \mathop \sum \limits_{i = 1}^{n} - x_{i} Sin\left( {\sqrt {\left| {x_{i} } \right|} } \right), \;\;\;\;\;\; - 500 \le x_{i} \le 500$$

**Figure 6:**
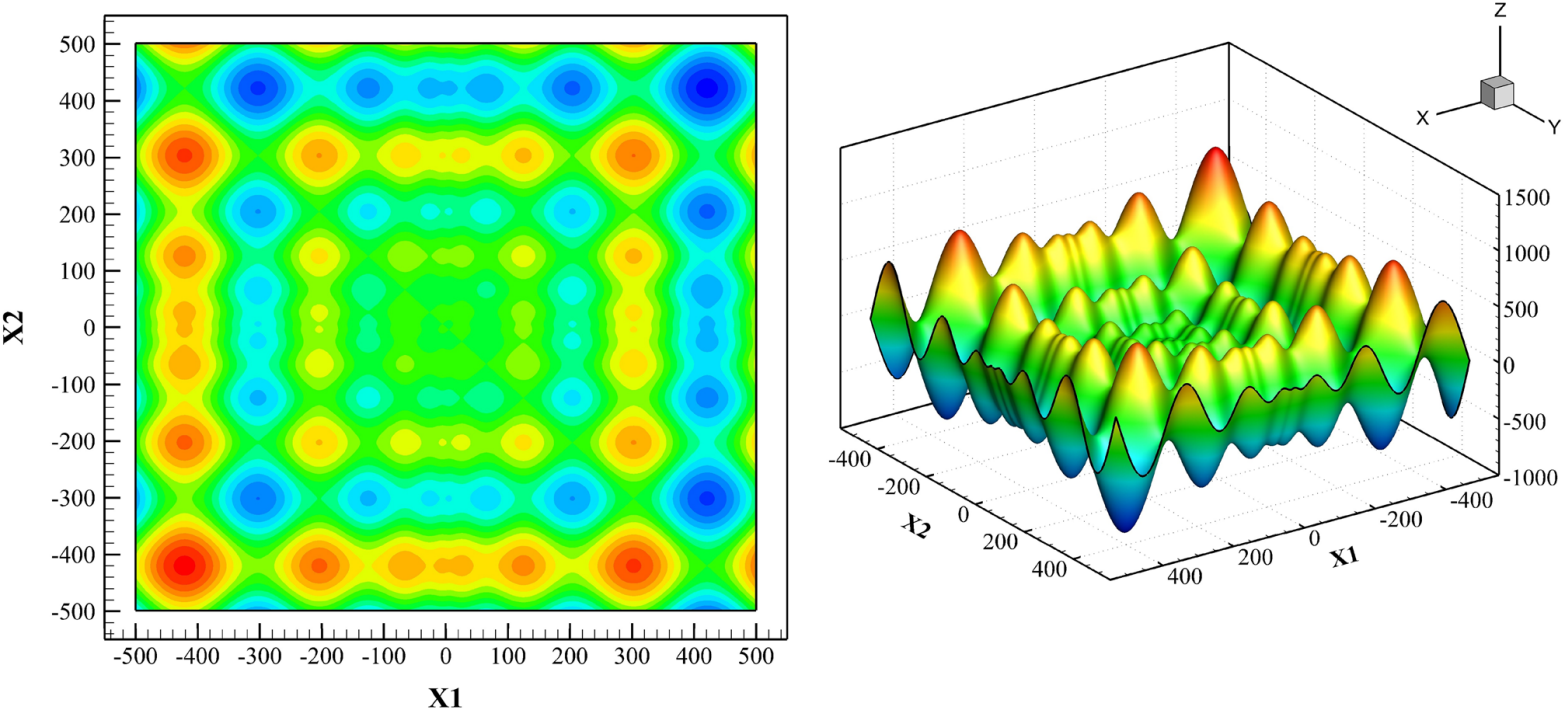
2D and 3D presentation of the two dimensional Schwefel function.

Here, n = 2. As seen in Fig. 6, the absolute minimum is located at ${x}_{i}=420.968$ and its value is $f\left(x\right)=-n\left(418.9829\right)$.

**Figure 7:**
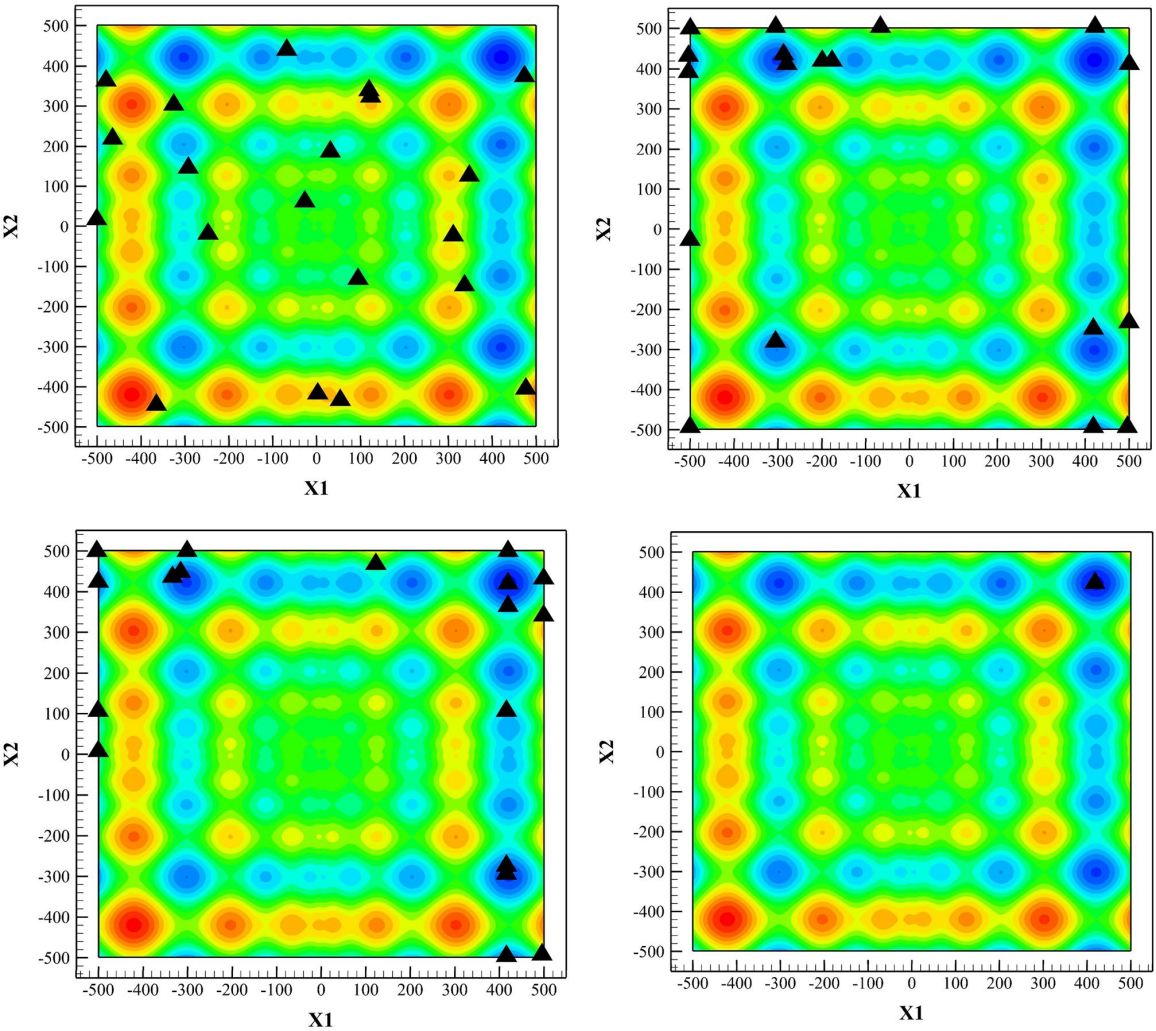
Using differential evolution to find the minimum of the objective function with ${N}_{p}=20$, $F=1.5$, ${C}_{r}=0.5$, top left to bottom right, respectively corresponding to generation No. 1, 50, 100, 250.

We use a population of 20, with a mutation factor $F=1.5$, with ${C}_{r}=0.5$. Distribution of the population for four different generations are shown in Fig. 7. As one observes, all members are converged towards the absolute minimum in the 250th generation, while the extremum is found in the 100th generation.

## Results and discussion

Here we use the optimization algorithm to find the optimum design for application of a single plasma actuator on a DU25 airfoil, with a chord of 1 m, a Reynolds number of 10^6^, and a free stream velocity of 14.61 m/s^61^. For a wind turbine, performance of the plamsa actuator at lower speeds is more important, since to harvest energy at low speeds, higher angles of attacks are necessary. Therefore, we use a Reynolds number of one million in this study. Four design variables are used here, i.e. the length of electrodes, the thickness of the dielectric, and its material. The optimization is done for 5 different voltages, and 10 different frequencies.

We use the plasma actuator to delay onset of stall in the aerodynamic performance. The induced wall jet generated by the actuator decreases the size and adverse effects of the separation bubble. We have investigated performance of this airfoil^45^ and have shown that the actuator has only a negligible effect on the performance before the flow separation. i.e. when the angle of attack is less than the stall angle (9°).

**Figure 8:**
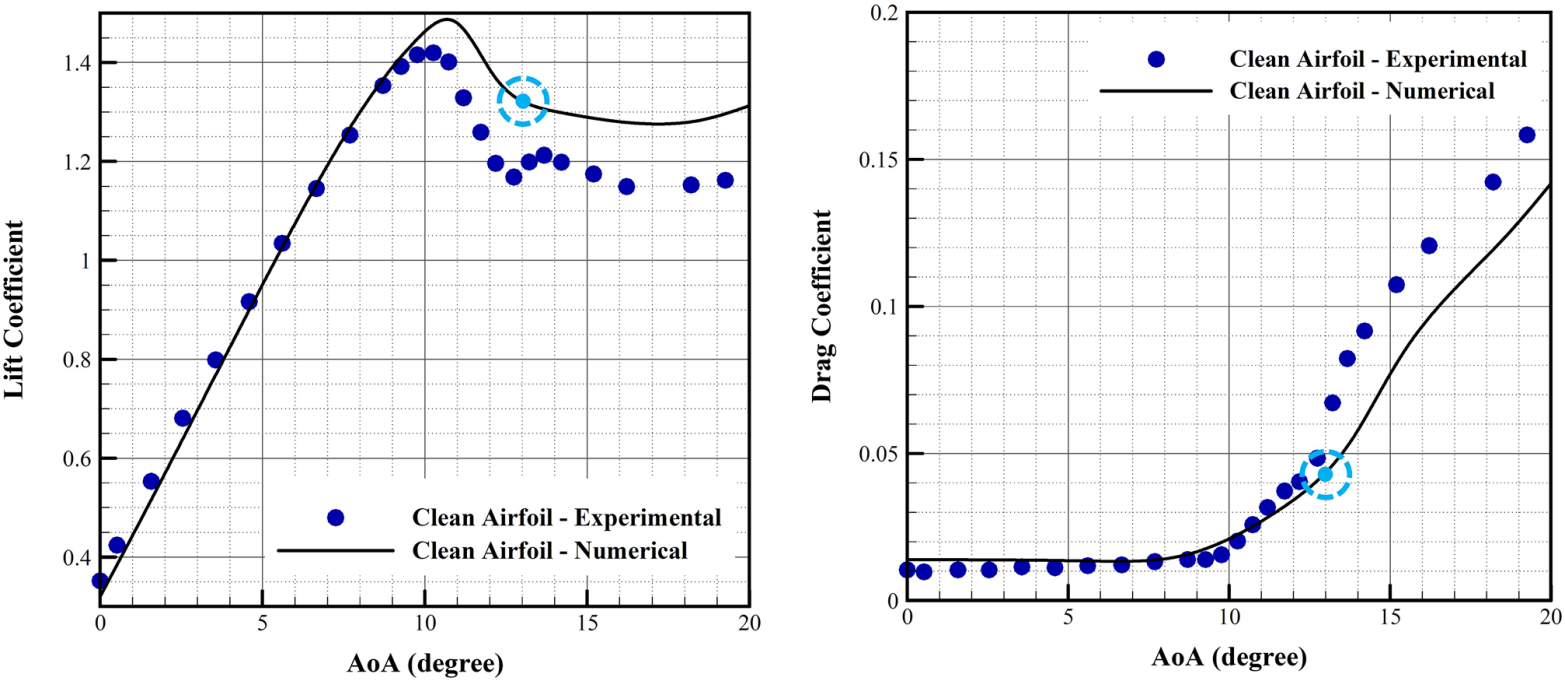
Lift and drag coefficients for DU25 airfoil (numerical and experimental^65^) at 14.61 m/s freestream velocity at different angles of attack. The optimization region is marked.

First we compare results of our simulation with the experimental data for the case of the DU25 airfoil without any actuator. Figure 8 shows the results. Results are fairly accurate enough for this study with comparison with the experimental data^65^. One observes that the maximum lift coefficient is achieved at 10° angle of attack, and then deep stall is observed at 13°. Therefore we use this 13° angle of attack for our optimization analysis. Figure 8 is not part of our validation process. It is shown to justify selection of 13° angle of attack for operating conditions. The difference between the experimental and numerical results are due to deficiency of our Navier stokes solver, which is not very accurate for highly separated flow dominated by large unsteady vortices, as it is the case for a deep stall condition. However, this deficiency will not affect our computations, since application of the plasma actuator, as described by authors^44,45^ significantly reduces the separation zone, and makes the vortices very small and steady, similar to the pre-stall conditions. Therefore this solver is accurate enough for our optimization problem.

**Figure 9:**
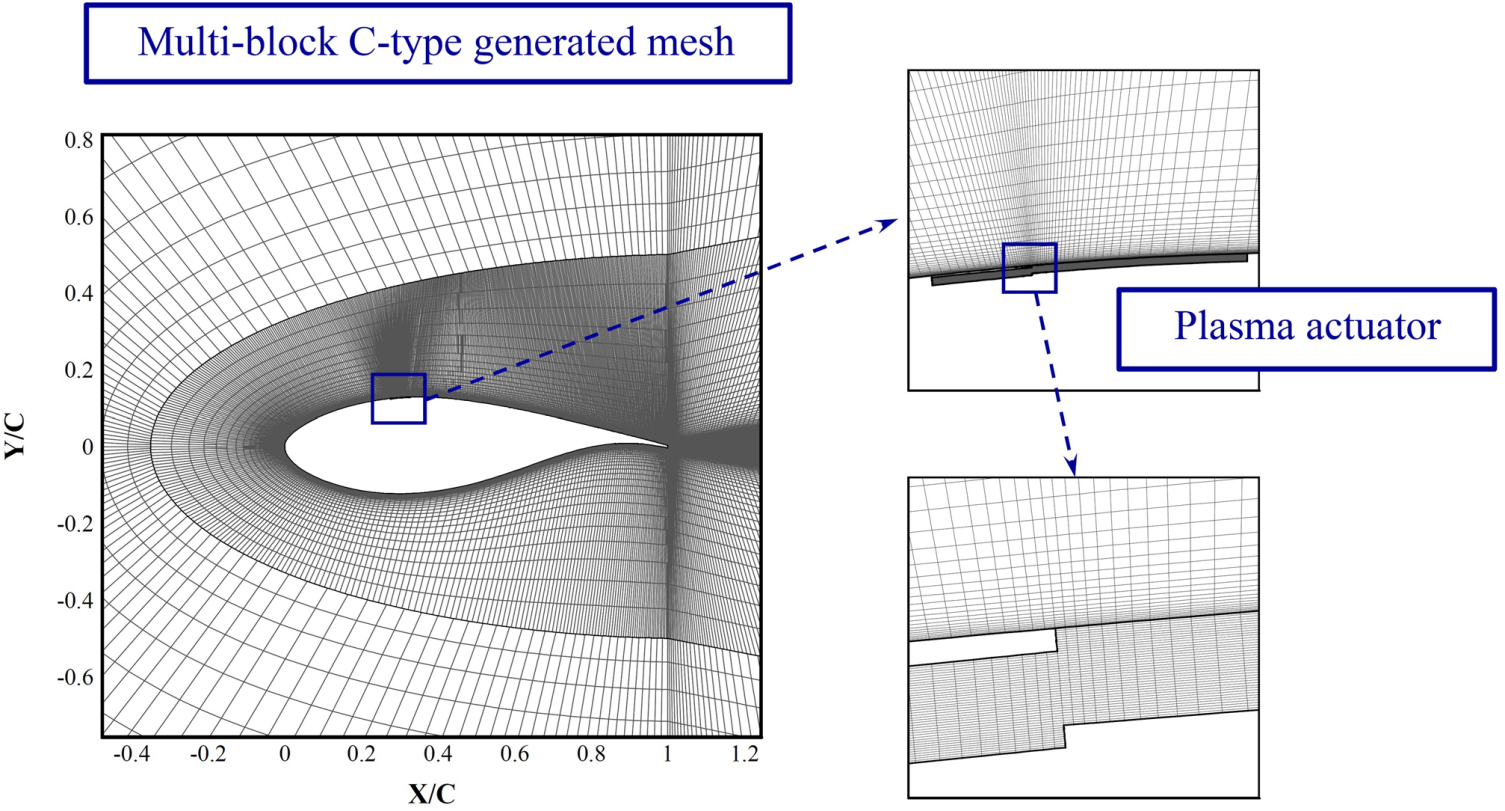
The numerical C grid generated around the DU25 airfoil, showing the grid in the dielectric region.

Figure 9 shows the C grid used for this study, when the base configuration is used. The generated mesh is very fine adjacent to the electrodes. Different studies show that a better location is close to the separation point [28 and 44] to control the wake size more effectively.

To select an optimization area for our design variables, we define a base configuration to search around it for an optimized solution. Previous studies [10, 44, and 45] show that for a configuration, which is called here the base configuration, with a permittivity of 4.2 ${C}^{2}/N{m}^{2}$, and thickness of 1 mm for the dielectric material, and length of 16 mm for the exposed electrode, and 30 mm for the embedded electrode, and a thickness of 0.15 mm for both electrodes, the aerodynamic performance of the this airfoil is greatly improved by application of the plasma actuator. The base voltage is 14 kV, with a frequency of 7 kHz. The actuator has shown to improve the lift coefficient by 53%, and the drag coefficient about 85%^44^. The separation point is also delayed to a section 33% of the chord length from the leading edge.

### The optimum of the embedded electrode length

**Figure 10:**
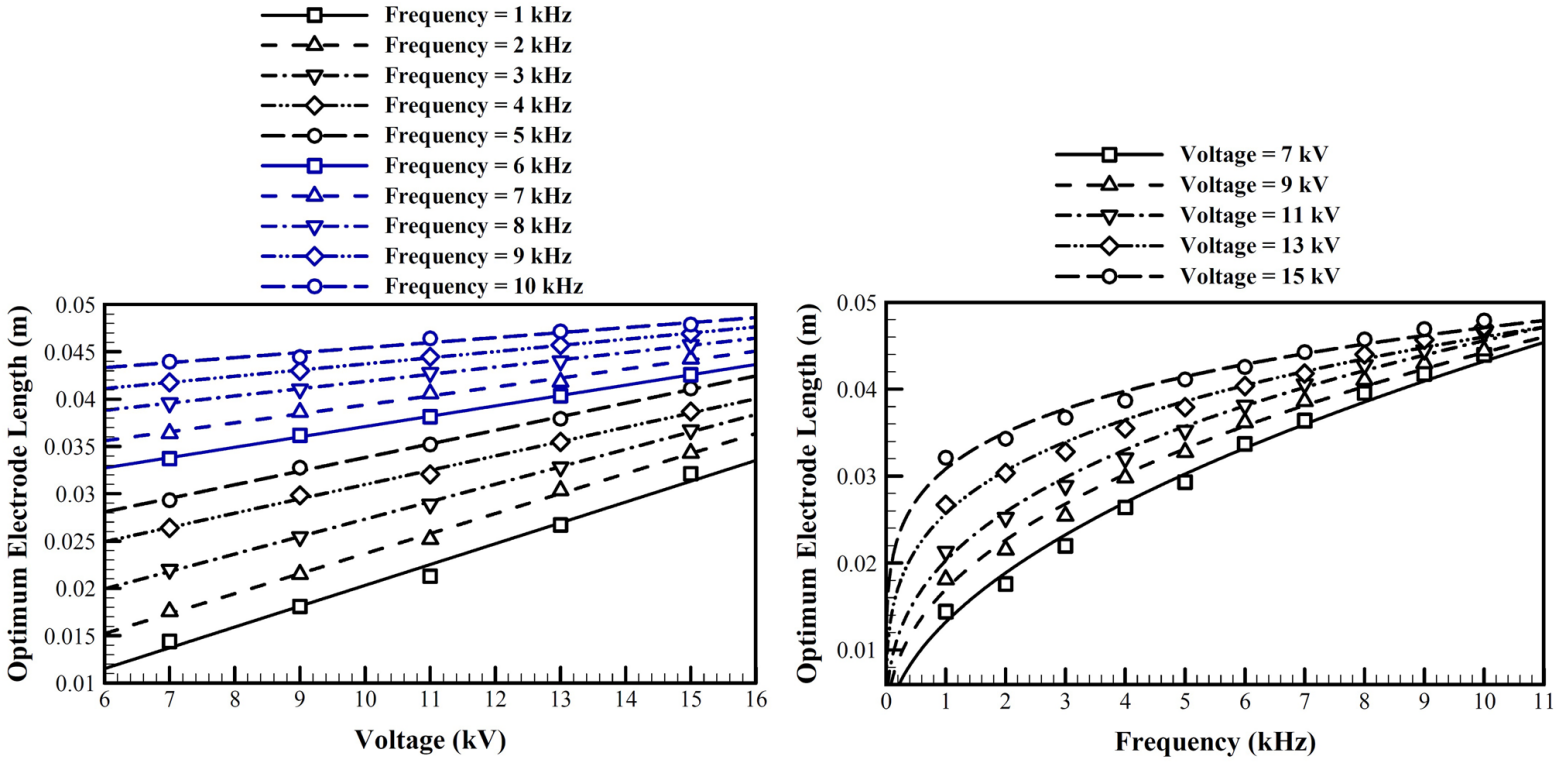
The optimum length of the embedded electrode for different voltages and frequencies.

Figure 10 shows the optimum length of the embedded electrode for five different voltages and ten different frequencies. Generally, the optimum length increases by both voltage and frequency, and for low voltages, optimum values of higher frequencies are more different from low frequencies. In fact we have a few physical phenomena competing in the flow field:

- Higher voltage, generates more ions, and induces a higher potential electric field, both effects are positive, but as the voltage is increased the incremental effect on ion generation decreases.
- Higher frequency helps in generation of more ions, but decreases the time for ion accumulation and generation of body force.
- Longer embedded electrode increases the high gradient region in the flow field, but decreases the potential gradient in the flow field. Also, longer electrodes allow for higher energy consumption and activating more ions and doing more work in their acceleration, which results in a higher body force.

One observes that when the frequency increases, its incremental effect on the optimized value decreases. This could be related to the fact that at a higher frequency there is not enough time for the charged particles on the electrode surface to accelerate, therefore different voltages act more similarly. Also, it is observed that at low frequencies, the optimized length is very sensitive to the voltage. This could be because of the fact that ions are accelerated over the embedded electrode for a half cycle, and a longer length help to accumulate more ions over the embedded electrode during the positive half cycle, and finally a greater body once is generated. As the length of the electrode increases more, the increased voltage has not any more significant effect as for shorter electrodes, i.e. very higher voltages are required to make the extra length as much effective. In other words the marginal effect of the increased voltage on the length of the plasma region decreases. The increase of length also increases the consumed power, and for high frequencies, may not be justified by the better performance of the actuator.

### The optimum dielectric permittivity

**Figure 11:**
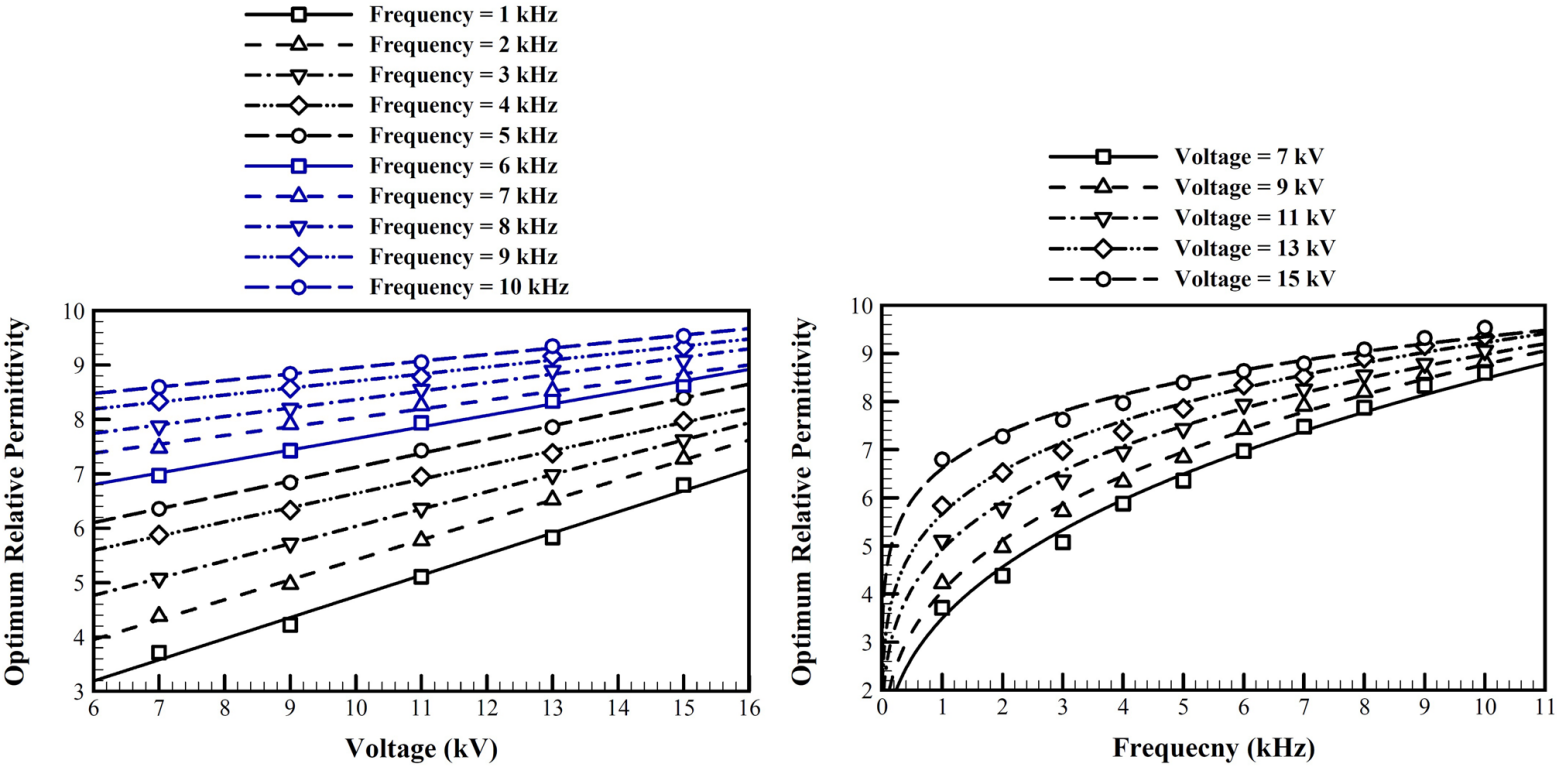
The optimum dielectric relative permittivity for different voltages and frequencies.

Figure 11 shows the optimized value for the permittivity of the dielectric material, for different voltages and frequencies. This figure is very similar to the optimized value of the length of the embedded electrode. Generally, increase of both frequency and voltage, increases the optimized value of the permittivity. In each working frequency, higher voltages provide a higher working capacity, which requires a higher permittivity and capacitance to generate a stronger induced wall jet. Similarly at any given voltage, a higher frequency provides a better capacity for generation of more ions and more consumption of energy, which requires a higher capacitance and permittivity. Similar to the discussion in the previous subsection, these marginal effects are weakened at higher frequencies and voltages.

### The optimum thickness of the dielectric and electrodes

Our study showed that the thickness of the dielectric material decreases the generated body force and the aerodynamic forces. We assumed that our physical model is not acceptable for very low thicknesses. However, the optimization always converged to the lower limit of its constraints. This study showed that when you increase this thickness, after some time, any further increase has very little effect on the body forces and the aerodynamic performance. Consequently the selection of the dielectric thickness or electrode thickness could not be based on the performance study, but shall be mostly related to fabrication technological limits or physical limits. Therefore we study that from the view point of aerodynamic performance, i.e. how thick the dielectric or the electrodes shall be, so that the performance does not change significantly.

**Figure 12:**
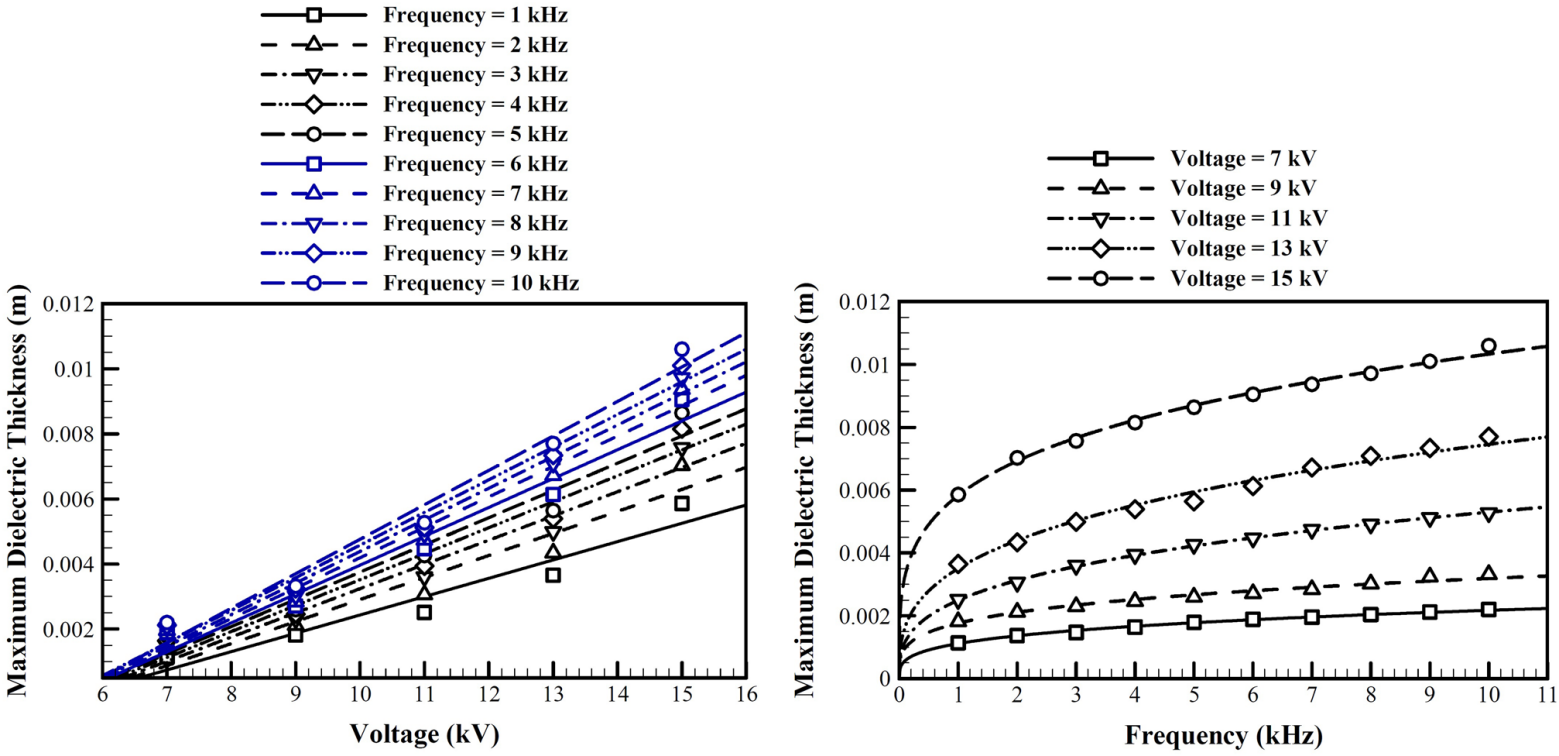
The maximum suitable dielectric thickness for different applied voltages and frequencies, at optimal conditions.

Figure 12 shows the maximum suitable dielectric thickness after which the change in the lift to drag ratio is less than 10% in the range studied in this study. The increase in the dielectric thickness decreases the gradient of the electrical potential, since it increases the distance between the embedded electrode and the flow field. This will decrease the body force and the acceleration of the ionized particles, which in its turn decreases the induced momentum to the boundary layer.

**Figure 13:**
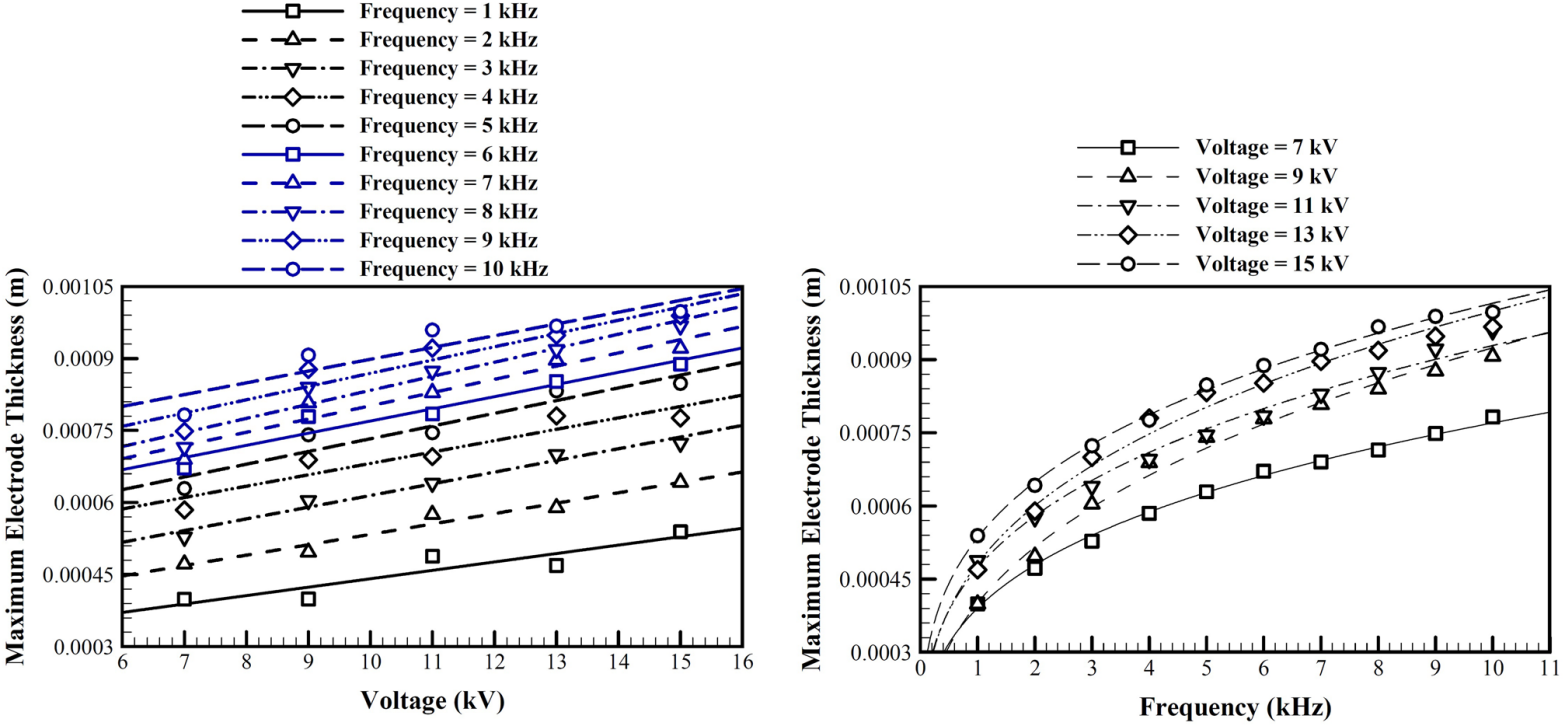
The maximum suitable electrode thickness for different applied voltages and frequencies, at optimal conditions.

Next we study the optimized values of the electrodes thicknesses. Our previous studies show that based on our model, any further decreases in their thickness improve the performance. Again, our model for capacitance of the dielectric barrier discharge which is used to find the plasma extent is not valid for very thin electrodes, and we believe that mostly the fabrication limits will define acceptable thicknesses. To find the electrode thickness after which results are not very sensitive to the electrode thickness, we performed a parametric study for different applied voltages and frequencies. The base configuration values are used for other conditions and parameters. Figure 13 shows the maximum suitable electrode thickness after which the decrease in the lift to drag ratio is more than 10%.

### Discussion

To make our results the most appropriate for an engineering design in wind turbine blades for better lift to drag ratio, here we introduce some simple design criteria to be used to achieve near optimum aerodynamic performance from plasma actuators. We introduce a power relation [Eq. (24)] with a base of applied frequency, $f$ in kHz, to be used for all four parameters used in this study, i.e. the dielectric material and thickness, and the embedded electrode length and thickness. We assume that the thickness of both electrodes are similar. We assume that the parameters a and b are linear functions of the applied voltage V, in volts. Table 2 shows the coefficients of these linear functions. The recommended dielectric and electrode thicknesses are the cut-off (maximum suitable) values introduced in Figs. 12 and 13.

24 $$\begin{gathered} P = af^{b} \hfill \\ a\left( V \right) = C_{1} V + C_{2} \hfill \\ b\left( V \right) = C_{3} V + C_{4} \hfill \\ \end{gathered}$$

**Table 2:** Coefficients of the recommended design formula.

Optimum parameter $(P)$	${C}_{1}$	${C}_{2}$	${C}_{3}$	${C}_{4}$
${l}_{e}^{Opt}$	0.0044	0.0082	− 0.0825	0.5917
${\varepsilon }_{rd}^{Opt}$	0.7842	2.5927	− 0.0592	0.4463
${t}_{e}^{cut-off}$	0	0.00046	− 0.0080	0.3331
${t}_{d}^{cut-off}$	0.0011	− 0.0004	0	0.3000

**Figure 14:**
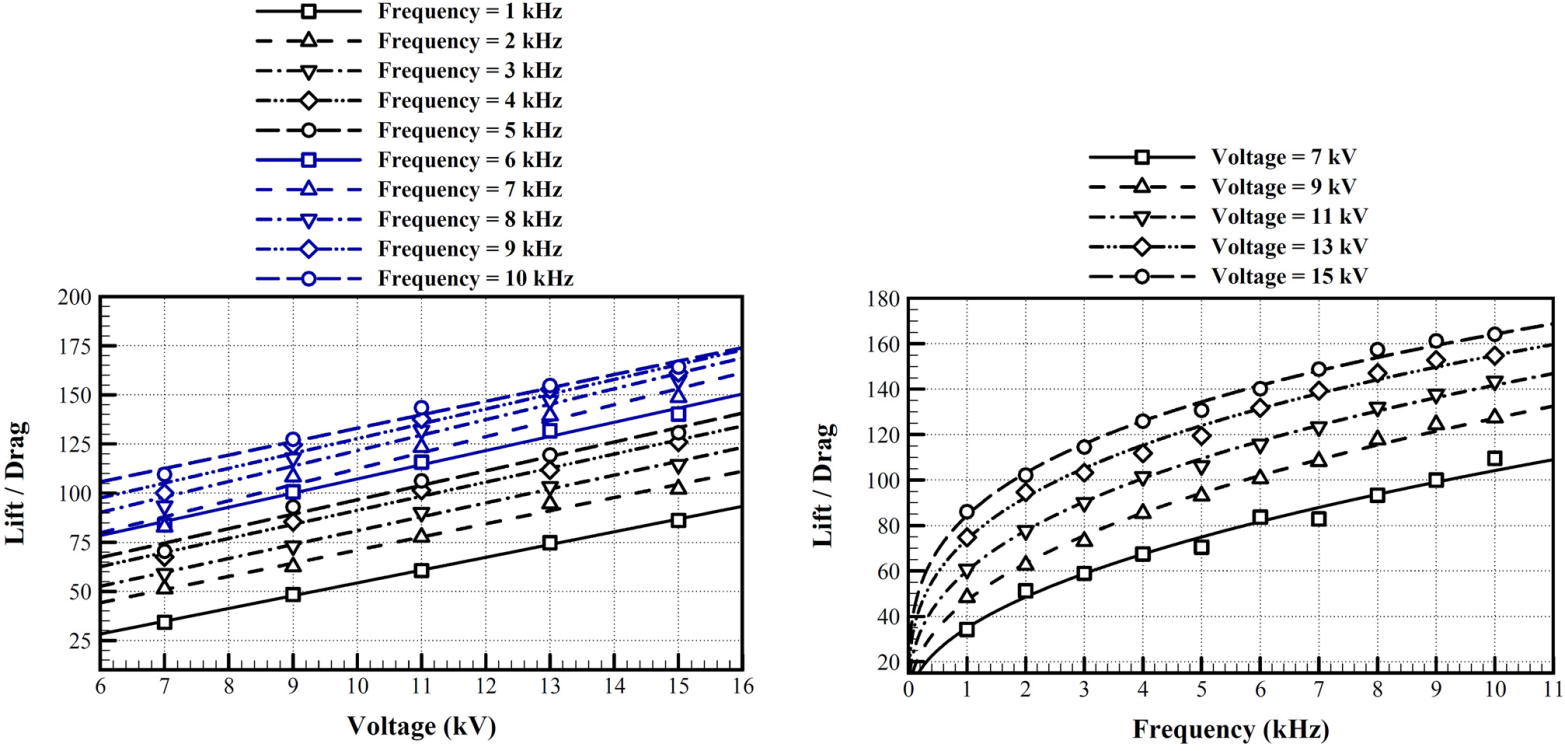
The optimized DU25 airfoil performance for different voltages and frequencies.

Finally, to show the effectiveness of the recommended algorithm to optimize the performance of a plasma actuator installed on a DU25 wind turbine blade, Fig. 14 shows the achieved aerodynamic performance, i.e. the lift to drag ratio, at the optimized conditions. A simple calculation shows that the consumed power is not significant in these cases. This figure shows that for the range of parameters studied here, both a higher voltage and a higher frequency improve the aerodynamic performance, although other technological limits may constraint the optimized solution. The incremental effects are higher at lower voltages and frequencies, and decreases by increase in the voltage and the frequency.

## Conclusion

Here we have optimized a plasma actuator installed on a DU25 airfoil of a wind turbine blade, for a better aerodynamic performance and energy harvesting. The differential evolution optimization algorithm was used, with an objective function of the ratio of drag to lift coefficient. Design variables are the length of the embedded electrode, the electrode thicknesses, and the permittivity of the dielectric material. Different voltages and frequencies were applied as varying parameters. The study was performed for wind velocity of 14.61 m/s at a highly loaded deep stall condition of 13° angle of attack. In this case, we have a leading edge separation with a huge separation bubble, covered most of the suction surface. The actuator was located at 30% chord distance from the leading edge for maximum flow control capability.

Results show that the optimized values of the embedded electrode length and the dielectric permittivity increases by increase in the applied voltage or frequency. We found that the exposed electrode length has little effect on performance. We observed that based on our model the electrode and dielectric thicknesses optimal values is always at our lower optimization constraint, but there is a threshold value after which further decrease has a negligible effect on aerodynamic performance. The threshold value increases by increase in the applied voltage and frequency. A new design relation is suggested which uses a power relation for all four design variables, to find the near optimum configuration. One also observes that at the recommended optimized configurations, the performance increases as a power function with a power of 0.5 respect to the voltage and frequency. We conclude that the proposed algorithm provides a basis for geometrical and design optimization of a DBD plasma actuator for application in wind turbine blades, to achieve the best aerodynamic performance for low speed winds and highly loaded blades.

## Nomenclature

$$E$$: Electric field, $$N/C$$
$$f$$: Frequency, $$Hz$$
${f}_{b}$: Body force vector, $N/{m}^{3}$
${l}_{e}$: Length of electrode, $m$
$${l}_{p}$$: Plasma extent, $$m$$
${n}_{i}$: Unit normal vector
$$P$$: Pressure, $$Pa$$
$$T$$: Temperature, $$K$$
$${t}_{d}$$: Dielectric thickness, $$m$$
$${t}_{e}$$: Electrode thickness, $$m$$
$${u}_{j}$$: Velocity component, $$m/s$$
$${V}_{app}$$: Applied voltage, $$Volt$$
$${V}_{bd}$$: Breakdown voltage, $$Volt$$
$$x,y$$: Coordinates
$${\varepsilon }_{r}$$: Relative permittivity
$${\lambda }_{d}$$: Debye length, $$m$$
$$\vartheta$$: Fluid viscosity, $${m}^{2}/s$$
$$\rho$$: Density, $$Kg/{m}^{3}$$
${\rho }_{c}$: Net charge density, $C/{m}^{3}$
$\phi$: Electric potential of external field, $Volt$
